# Elderly Fall Detection Systems: A Literature Survey

**DOI:** 10.3389/frobt.2020.00071

**Published:** 2020-06-23

**Authors:** Xueyi Wang, Joshua Ellul, George Azzopardi

**Affiliations:** ^1^Department of Computer Science, Bernoulli Institute for Mathematics, Computer Science and Artificial Intelligence, University of Groningen, Groningen, Netherlands; ^2^Computer Science, Faculty of Information & Communication Technology, University of Malta, Msida, Malta

**Keywords:** fall detection, Internet of Things (IoT), information system, wearable device, ambient device, sensor fusion

## Abstract

Falling is among the most damaging event elderly people may experience. With the ever-growing aging population, there is an urgent need for the development of fall detection systems. Thanks to the rapid development of sensor networks and the Internet of Things (IoT), human-computer interaction using sensor fusion has been regarded as an effective method to address the problem of fall detection. In this paper, we provide a literature survey of work conducted on elderly fall detection using sensor networks and IoT. Although there are various existing studies which focus on the fall detection with individual sensors, such as wearable ones and depth cameras, the performance of these systems are still not satisfying as they suffer mostly from high false alarms. Literature shows that fusing the signals of different sensors could result in higher accuracy and lower false alarms, while improving the robustness of such systems. We approach this survey from different perspectives, including data collection, data transmission, sensor fusion, data analysis, security, and privacy. We also review the benchmark data sets available that have been used to quantify the performance of the proposed methods. The survey is meant to provide researchers in the field of elderly fall detection using sensor networks with a summary of progress achieved up to date and to identify areas where further effort would be beneficial.

## 1. Introduction

More than nine percent of the population of China was aged 65 or older in 2015 and within 20 years (2017–2037) it is expected to reach 20%^1^. According to the World Health Organization (WHO), around 646 k fatal falls occur each year in the world, the majority of whom are suffered by adults older than 65 years (WHO, 2018). This makes it the second reason for unintentional injury death, followed by road traffic injuries. Globally, falls are a major public health problem for the elderly. Needless to say, the injuries caused by falls that elderly people experience have many consequences to their families, but also to the healthcare systems and to the society at large.

**Figure 1:**
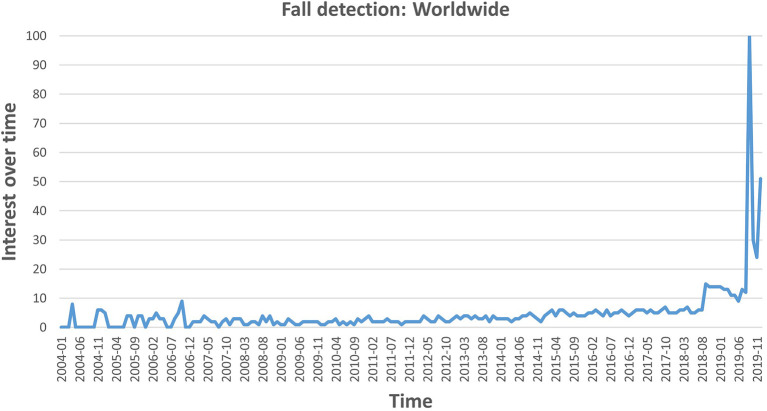
Interest of fall detection over time, from January 2004 to December 2019. The data is taken from Google Trends with the search topic “fall detection.” The values are normalized with the maximum interest, such that the highest interest has a value of 100.

As illustrated in Figure 1, Google Trends^2^ show that fall detection has drawn increasing attention from both academia and industry, especially in the last couple of years, where a sudden increase can be observed. Moreover, on the same line, the topic of fall-likelihood prediction is very significant too, which is coupled with some applications focused on prevention and protection.

El-Bendary et al. (2013) reviewed the trends and challenges of elderly fall detection and prediction. Detection techniques are concerned with recognizing falls *after* they occur and trigger an alarm to emergency caregivers, while predictive methods aim to forecast fall incidents *before* or *during* their occurrence, and therefore allow immediate actions, such as the activation of airbags.

**Figure 2:**
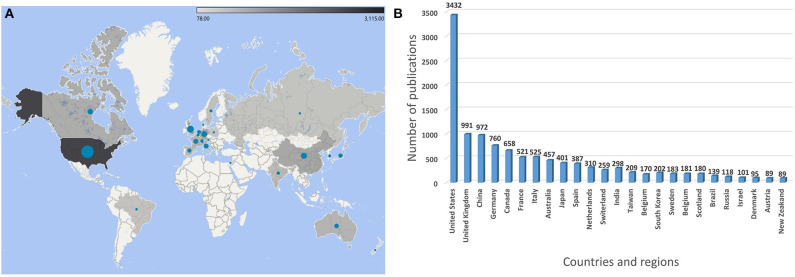
**(A)** A map and **(B)** a histogram of publications on fall detection by countries and regions from 1945 to 2020.

During the past decades, much effort has been put into these fields to improve the accuracy of fall detection and prediction systems as well as to decrease the false alarms. Figure 2 shows the top 25 countries in terms of the number of publications about fall detection from the year 1945 to 2020. Most of the publications originate from the United States, followed by England, China, and Germany, among others. The data indicates that developed countries invest more in conducting research in this field than others. Due to higher living standards and better medical resources, people in developed countries are more likely to have longer life expectancy, which results in a higher aging population in such countries (Bloom et al., 2011).

In this survey paper, we provide a holistic overview of fall detection systems, which is aimed for a broad readership to become abreast with the literature in this field. Besides fall detection modeling techniques, this review covers other topics including issues pertaining to data transmission, data storage and analysis, and security and privacy, which are equally important in the development and deployment of such systems.

The other parts of the paper are organized as follows. In section 2, we start by introducing the types of fall and reviewing other survey papers to illustrate the research trend and challenges up to date, followed by a description of our literature search strategy. Next, in section 3 we introduce hardware and software components typically used in fall detection systems. Sections 4 and 5 give an overview of fall detection methods that rely on both individual or a collection of sensors. In section 6, we address issues of security and privacy. Section 7 introduces projects and applications of fall detection. In section 8, we provide a discussion about the current trends and challenges, followed by a discussion on challenges, open issues, and other aspects on future directions. Finally, we provide a summary of the survey and draw conclusions in section 9.

## 2. Types of Falls and Previous Reviews on Elderly Fall Detection

### 2.1. Types of Falls

The impact and consequences of a fall can vary drastically depending upon various factors. For instance, falling whilst either walking, standing, sleeping or sitting on a chair, share some characteristics in common but also have significant differences between them.

In El-Bendary et al. (2013), the authors group the types of falls in three basic categories, namely *forward, lateral*, and *backward*. Putra et al. (2017) divided falls into a broader set of categories, namely *forward, backward, left-side, right-side, blinded-forward*, and *blinded-backward*, and in the study by Chen et al. (2018) falls are grouped in more specific categories including *fall lateral left lie on the floor, fall lateral left and sit up from floor, fall lateral right and lie on the floor, fall lateral and left sit up from the floor, fall forward and lie on the floor*, and *fall backward and lie on the floor*.

Besides the direction one takes whilst falling another important aspect is the duration of the fall, which may be influenced by age, health and physical condition, along with any consequences of activities that the individual was undertaking. Elderly people may suffer from longer duration of falls, because of motion with low speed in the activity of daily living. For instance, in fainting or chest pain related episodes an elderly person might try to rest by a wall before lying on the floor. In other situations, such as injuries due to obstacles or dangerous settings (e.g., slanting or uneven pavement or surfaces), an elderly person might fall abruptly. The age and gender of the subject also play a role in the kinematics of falls.

The characteristics of different types of falls are not taken into consideration in most of the work on fall detection surveyed. In most of the papers to date, data sets typically contain falls that are simulated by young and healthy volunteers and do not cover all types of falls mentioned above. The resulting models from such studies, therefore, do not lead to models that generalize well enough in practical settings.

### 2.2. Review of Previous Survey Papers

There are various review papers that give an account of the development of fall detection from different aspects. Due to the rapid development of smart sensors and related analytical approaches, it is necessary to re-illustrate the trends and development frequently. We choose the most highly cited review papers, from 2014 to 2020, based on Google Scholar and Web of Science, and discuss them below. These selected review papers demonstrate the trends, challenges, and development in this field. Other significant review papers before 2014 are also covered in order to give sufficient background of earlier work.

Chaudhuri et al. (2014) conducted a systematic review of fall detection devices for people of different ages (excluding children) from several perspectives, including background, objectives, data sources, eligibility criteria, and intervention methods. More than 100 papers were selected and reviewed. The selected papers were divided into several groups based on different criteria, such as the age of subjects, method of evaluation and devices used in detection systems. They noted that most of the studies were based on synthetic data. Although simulated data may share common features with real falls, a system trained on such data cannot reach the same reliability of those that use real data.

In another survey, Zhang et al. (2015) focused on vision-based fall detection systems and their related benchmark data sets, which have not been discussed in other reviews. Vision-based approaches of fall detection were divided into four categories, namely individual single RGB cameras, infrared cameras, depth cameras, and 3D-based methods using camera arrays. Since the advent of depth cameras, such as Microsoft Kinect, fall detection with RGB-D cameras has been extensively and thoroughly studied due to the inexpensive price and easy installation. Systems which use calibrated camera arrays also saw prominent uptake. Because such systems rely on many cameras positioned at different viewpoints, challenges related to occlusion are typically reduced substantially, and therefore result in less false alarm rates. Depth cameras have gained particular popularity because unlike RGB camera arrays they do not require complicated calibration and they are also less intrusive of privacy. Zhang et al. (2015) also reviewed different types of fall detection methods, that rely on the activity/inactivity of the subjects, shape (width-to-height ratio), and motion. While that review gives a thorough overview of vision-based systems, it lacks an account of other fall detection systems that rely on non-vision sensors such as wearable and ambient ones.

Further to the particular interest in depth cameras, Cai et al. (2017) reviewed the benchmark data sets acquired by Microsoft Kinect and similar cameras. They reviewed 46 public RGB-D data sets, 20 of which are highly used and cited. They compared and highlighted the characteristics of all data sets in terms of their suitability to certain applications. Therefore, the paper is beneficial for scientists who are looking for benchmark data sets for the evaluation of new methods or new applications.

Based on the review provided by Chen et al. (2017a), individual depth cameras and inertial sensors seem to be the most significant approaches in vision- and non-vision-based systems, respectively. In their review, the authors concluded that fusion of both types of sensor resulted in a system that is more robust than a system relying on one type of sensor.

The ongoing and fast development in electronics have resulted in more miniature and cheaper electronics. For instance, the survey by Igual et al. (2013) noted that low-cost cameras and accelerometers embedded in smartphones may offer the most sensible technological choice for the investigation of fall detection. Igual et al. (2013) identified two main trends on how research is progressing in this field, namely the use of vision and smartphone-based sensors that give input and the use of machine learning for the data analysis. Moreover, they reported the following three main challenges: (i) real-world deployment performance, (ii) usability, and (iii) acceptance. Usability refers to how practical the elderly people find the given system. Because of the issue of privacy and intrusive characteristics of some sensors, there is a lack of acceptance for the elderly to live in an environment monitored by sensors. They also pointed out several issues which need to be taken into account, such as smartphone limitations (e.g., people may not carry smartphones all the time with them), privacy concerns, and the lack of benchmark data sets of realistic falls.

The survey papers mentioned above focus mostly on the different types of sensors that can be used for fall detection. To the best of our knowledge, there are no literature surveys that provide a holistic review of fall detection systems in terms of data acquisition, data analysis, data transport and storage, sensor networks and Internet of Things (IoT) platforms, as well as security and privacy, which are significant in the deployment of such systems.

### 2.3. Key Results of Pioneering Papers

In order to illustrate a timeline of fall detection development, in this section we focus on the key and pioneering papers. Through manual filtering of papers using the web of science, one can find the trendsetting and highly cited papers in this field. By analyzing retrieved articles using citespace one can find that fall detection research first appeared in the 1990s, beginning with the work by Lord and Colvin (1991) and Williams et al. (1998). A miniature accelerometer and microcomputer chip embedded in a badge was used to detect falls (Lord and Colvin, 1991), while Williams et al. (1998) applied a piezoelectric shock sensor and a mercury tilt switch which monitored the orientation of the body to detect falls. At first, most studies were based on accelerometers including the work by Bourke et al. (2007). In their work, they compared which of the trunk and thigh offer the best location to attach the sensor. Their results showed that a person's trunk is a better location in comparison to the thigh, and they achieved 100% specificity with a certain threshold value with a sensor located in the trunk. This method was the state-of-the-art at the time, which undoubtedly supported it in becoming the most highly cited paper in the field.

At the time the trend was to use individual sensors for detection, within which another key paper by Bourke and Lyons (2008) was proposed to explore the problem at hand by using a single gyroscope that measures three variables, namely angular velocity, angular acceleration, and the change in the subject's trunk-angle. If the values of these three variables in a particular instance are above some empirically determined thresholds, then that instance is flagged as a fall. Three thresholds were set to distinguish falls from non-falls. Falls are detected when the angular velocity of a subject is greater than the fall threshold, and the angular acceleration of the subject is greater than the second fall threshold, and the change in the trunk-angle of the subject is greater than the third fall threshold. They reported accuracy of 100% on a data set with only four kinds of falls and 480 movements simulated by young volunteers. However, for those classifiers, which are based solely on either accelerometers or gyroscopes, are argued to suffer from insufficient robustness (Tsinganos and Skodras, 2018). Later, Li et al. (2009) investigated fusion of gyroscope and accelerometer data for the classification of falls and non-falls. In their work, they demonstrated how a fusion based approach resulted in a more robust classification. For instance, it could distinguish falls more accurately from certain fall-like activities, such as sitting down quickly and jumping, which is hard to detect using a single accelerometer. This work had inspired further research on sensor fusion. These two types of sensors can nowadays be found in all smart phones (Zhang et al., 2006; Dai et al., 2010; Abbate et al., 2012).

Besides the two non-vision based types of sensors mentioned above, vision-based sensors, such as surveillance cameras, and ambience-based, started becoming an attractive alternative. Rougier et al. (2011b) proposed a shape matching technique to track a person's silhouette through a video sequence. The deformation of the human shape is then quantified from the silhouettes based on shape analysis methods. Finally, falls are classified from normal activities using a Gaussian mixture model. After surveillance cameras, depth cameras also attracted substantial attention in this field. The earliest research which applied Time-of-Flight (TOF) depth camera was conducted in 2010 by Diraco et al. (2010). They proposed a novel approach based on visual sensors, which does not require landmarks, calibration patterns or user intervention. A ToF camera is, however, expensive and has low image resolution. Following that, the Kinect depth camera was first used in 2011 by Rougier et al. (2011a). Two features, human centroid height and velocity of body, were extracted from depth information. A simple threshold based algorithm was applied to detect falls and an overall success rate of 98.7% was achieved.

After the introduction of Kinect by Microsoft, there was a large shift in research from accelerometers to depth cameras. Accelerometers and depth cameras have become the most popular individual and combined sensors (Li et al., 2018). The combination of these two sensors achieved a substantial improvement when compared to the individual use of the sensors separately.

### 2.4. Strategy of the Literature Search

We use two databases, namely Web of Science and Google Scholar, to search for relevant literature. Since the sufficient advancements have been made at a rapid pace recently, searches included articles that were published in the last 6 years (since 2014). We also consider, all survey papers that were published on the topic of fall detection. Moreover, we give an account of all relevant benchmark data sets that have been used in this literature.

For the keywords “fall detection”, 4,024 and 575,000 articles were found for the above two mentioned databases, respectively, since 2014. In order to narrow down our search to the more relevant articles we compiled a list of the most frequently used keywords that we report in Table 1.

**Table 1:** The most frequently used keywords in the topic of fall detection.

**Wearable sensor**	**Visual sensor**	**Ambient sensor**	**Sensor fusion**
Fall detection	Fall detection	Fall detection	Fall detection
Falls	Falls	Falls	Falls
Fall accident	Fall accident	Fall accident	Fall accident
Machine learning	Machine learning	Machine learning	Machine learning
Deep learning	Deep learning	Deep learning	Deep learning
Reinforcement learning	Reinforcement learning	Reinforcement learning	Reinforcement learning
Body area networks	Multiple camera	Ambient sensor	Health monitoring
Wearable	Visual	Ambient	Sensor fusion
Worn	Vision-based	Ambience	Sensor network
Accelerometer	Kinect	RF-sensing	Data fusion
Gyroscope	Depth camera	WiFi	Multiple sensors
Biosensor	Video surveillance	Radar	Camera arrays
Smart watch	RGB camera	Cellular	Decision fusion
Gait	Infrared camera	Vibration	Anomaly detection
Wearable based	Health- monitoring	Ambience-based	IoT

*They are manually classified into four categories*.

**Figure 3:**
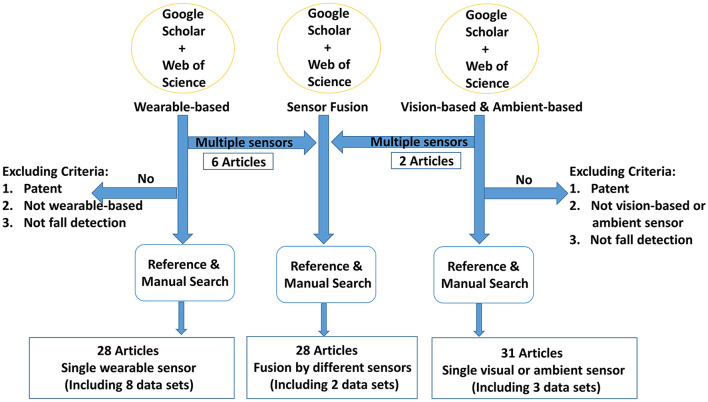
Illustration of the literature search strategy. The wearable-based queries in Table 2 return 28 articles. The vision- and ambient-based queries return 31 articles, and the sensor fusion queries return 28 articles.

We use the identified keywords above to generate the queries listed in Table 2 in order to make the search more specific to the three classes of sensors that we are interested in. For the retrieved articles, we discuss their contributions and keep only those that are truly relevant to our survey paper. For instance, articles that focus on rehabilitation after falls, and causes of falls, among others, are filtered out manually. This process, which is illustrated in Figure 3, ends up with a total of 87 articles, 13 of which describe benchmark data sets.

**Table 2:** Search queries used in Google Scholar and Web of Science for the three types of sensor and sensor fusion.

**Sensor type**	**Query**
Wearable-based	(Topic): ((“Fall detection" OR “Fall” OR “Fall accident”) AND (“Wearable” OR “Worn” OR “Accelerometer” OR “Machine learning” OR “Deep learning” OR “Reinforcement learning”) NOT “Survey” NOT “Review” NOT “Kinect” NOT “Video” NOT “Infrared” NOT “Ambient”)
Vision-based	(Topic): ((“Fall detection” OR “Falls” OR “Fall accident”) AND (“Video” OR “Visual” OR “Vision-based” OR “Kinect” OR “Depth camera” OR “Video surveillance” OR “RGB camera” OR “Infrared camera” OR “Monocular camera” OR “Machine learning” OR “Deep learning” OR “Reinforcement learning”) NOT “Wearable” NOT “Ambient”)
Ambient-based	(Topic): ((“Fall detection” OR “Falls” OR “Fall accident”) AND (“Ambient” OR “Ambient-based” OR “Ambience-based” OR “RF-sensing” OR “WiFi” OR “Cellular” OR “vibration” OR “Ambience” OR “Radar” OR “Machine learning” OR “Deep learning” OR “Reinforcement learning”) NOT “Wearable” NOT “vision”)
Sensor Fusion	(Topic): ((“Fall detection” OR “Falls” OR “Falls accident”) AND (“Health monitoring” OR “Multiple sensors” OR “Sensor fusion” OR “Sensor network” “Data fusion” OR “IoT” OR “Camera arrays” OR “Decision fusion” OR “Health monitoring” OR “Fusion” OR “Multiple sensors” OR “Machine learning” OR “Deep learning” OR “Reinforcement learning”))

## 3. Hardware and Software Components Involved in a Fall Detection System

**Figure 4:**
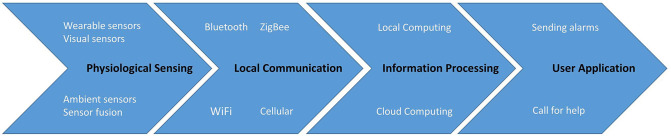
The main components typically present within fall detection system architectures include the illustrated sequence of four layers. Data is collected in the physiological sensing layer, transferred through the local communication layer, then it is analyzed in the information processing layer, and finally the results are presented in the user application layer.

Most of the research of fall detection share a similar system architecture, which can be divided into four layers, namely Physiological Sensing Layer (PSL), Local Communication Layer (LCL), Information Processing Layer (IPL), and User application Layer (UAL), as suggested by Ray (2014) and illustrated in Figure 4.

PSL is the fundamental layer that contains various (smart) sensors used to collect physiological and ambient data from the persons being monitored. The most commonly used sensors nowadays include accelerometers that sense acceleration, gyroscopes that detect angular velocity, and magnetometers which sense orientation. Video surveillance cameras, which provide a more traditional means of sensing human activity, are also often used but are installed in specific locations, typically with fixed fields of views. More details about PSL are discussed in sections 4.1 and 5.1.

The next layer, namely LCL, is responsible for sending the sensor signals to the upper layers for further processing and analysis. This layer may have both wireless and wired methods of transmission, connected to local computing facilities or to cloud computing platforms. LCL typically takes the form of one (or potentially more) communication protocols, including wireless mediums like cellular, Zigbee, Bluetooth, WiFi, or even wired connections. We provide more details on LCL in sections 4.2 and 5.2.

IPL is a key component of the system. It includes hardware and software components, such as micro-controller, to analyze and transfer data from PSL to higher layers. In terms of software components, different kinds of algorithms, such as threshold, conventional machine learning, deep learning, and deep reinforcement learning are discussed in sections 4.3, 5.3, and 8.1.

Finally, the UAL concerns applications that assist the users. For instance, if a fall is detected in the IPL, a notification can first be sent to the user and if the user confirms the fall or does not answer, an alarm is sent to the nearest emergency caregivers who are expected to take immediate action. There are plenty of other products like Shimmer and AlertOne, which have been deployed as commercial applications to users. We also illustrate other different kinds of applications in section 7.

## 4. Fall Detection Using Individual Sensors

### 4.1. Physiological Sensing Layer (PSL) of Individual Sensors

As mentioned above, fall detection research applied either a single sensor or fusion by multiple sensors. The methods of collecting data are typically divided into four main categories, namely individual wearable sensors, individual visual sensors, individual ambient sensors and data fusion by sensor networks. Whilst some literature groups visual and ambient sensors together we treat them as two different categories in this survey paper due to visual sensors becoming more prominent as a detection method with the advent of depth cameras (RGBD), such as the Kinect.

#### 4.1.1. Individual Wearable Sensors

Falls may result in key physiological variations of the human body, which provide a criterion to detect a fall. By measuring various human body related attributes using accelerometers, gyroscopes, glucometers, pressure sensors, ECG (Electrocardiography), EEG (Electroencephalography), or EOG (Electromyography), one can detect anomalies within subjects. Due to the advantages of mobility, portability, low cost, and availability, wearable devices are regarded as one of the key types of sensors for fall detection and have been widely studied. Numerous studies have been conducted to investigate wearable devices, which are regarded as a promising direction to study fall detection and prediction.

Based on our search criteria and filtering strategy (Tables 1, 2), 28 studies, including eight papers focusing on public data sets, focusing on fall detection by individual wearable devices are selected and described to illustrate trends and challenges of fall detection during the past 6 years. Some conclusions can be drawn based on the literature during the past 6 years in comparison to the studies before 2014. From Table 3, we note that studies applying accelerometers account for a large percentage of research in this field. To the best of our knowledge, only Xi et al. (2017) deployed electromyography to detect falls, and 19 out of 20 papers applied an accelerometer to detect falls. Although the equipment used, such as Shimmer nodes, smartphones, and smart watches, often contain other sensors like gyroscopes and magnetometers, these sensors were not used to detect falls. Bourke et al. (2007) also found that accelerometers are regarded as the most popular sensors for fall detection mainly due to its affordable cost, easy installation and relatively good performance.

**Table 3:** Fall detection using individual wearable devices from 2014 to 2020.

**References**	**Sensor**	**Location**	**No. subjects (age)**	**Data sets**	**Algorithms**	**Equipment**	**Alarm**
Saleh and Jeannès (2019)	Accelerometer	Waist	23 (19–30), 15 (60–75)	Simulated	SVM	N/A	N
Zitouni et al. (2019)	Accelerometer	Sole	6 (N/A)	Simulated	Threshold	Smartsole	N/A
Thilo et al. (2019)	Accelerometer	Torso	15 (mean = 81)	N/A	N/A	N/A	Y
Wu et al. (2019)	Accelerometer	Chest and Thigh	42 (N/A), 36 (N/A)	Public (Simulated)	Decision tree	Smartwatch (Samsung watch)	N/A
Sucerquia et al. (2018)	Accelerometer	Waist	38 (N/A)	Public data sets			
Chen et al. (2018)	Accelerometer	Leg (pockets)	10 (20–26)	N/A	ML(SVM)	Smartphones	Y
Putra et al. (2017)	Accelerometer	Waist	38 (N/A), 42 (N/A)	Public data sets	ML	N	N/A
Khojasteh et al. (2018)	Accelerometer	N/A	17 (18–55), 6 (N/A), 15 (mean = 66.4)	Public (Simulated)	Threshold/ML	N/A	N/A
de Araújo et al. (2018)	Accelerometer	Wrist	1 (30)	N/A	Threshold	Smartwatch	N/A
Djelouat et al. (2017)	Accelerometer	Waist	N/A	Collected by authors (Simulated)	ML	Shimmer-3	Y
Aziz et al. (2017)	Accelerometer	Waist	10 (mean = 26.6)	Collected by authors (Simulated)	Threshold/ML	Accelerometers (Opal model, APDM Inc)	N
Kao et al. (2017)	Accelerometer	Wrist	N/A	Collected by authors (Simulated)	ML	ZenWatch(ASUS)	Y
Islam et al. (2017)	Accelerometer	Chest (pocket)	7 (N/A)	N/A	Threshold	Smartphone	N/A
Xi et al. (2017)	Electro-myography (sEMG)	Ankle, Leg	3 (24–26)	Collected by authors (Simulated)	ML	EMGworks 4.0 (DelSys Inc.)	N
Chen et al. (2017b)	Accelerometer	Lumbar, Thigh	22 (mean = 69.5)	Public data sets (Real)	ML	N/A	N/A
Chen et al. (2017b)	Accelerometer	Chest, Waist, Arm, Hand	N/A	Collected by authors (Simulated)	Threshold	N/A	Y
Medrano et al. (2017)	Accelerometer	N/A	10 (20–42)	Public (Simulated)	ML	Smartphones	N
Shi et al. (2016)	Accelerometer	N/A	10 (mean = 25)	N/A	Threshold	Smartphone	N/A
Wu et al. (2015)	Accelerometer	Waist	3 (23, 42, 60)	Collected by authors (Simulated)	Threshold	ADXL345 Accelerometer(ADI)	Y
Mahmud and Sirat (2015)	Accelerometer	Waist	13 (22–23)	Collected by authors (Simulated)	Threshold	Shimmer	N/A

*ML is the abbreviation of Machine Learning*.

Although smartphones have gained attention for studying falls, the underlying sensors of systems using them are still accelerometers and gyroscopes (Shi et al., 2016; Islam et al., 2017; Medrano et al., 2017; Chen et al., 2018). Users are more likely to carry smartphones all day rather than extra wearable devices, so smartphones are useful for eventual real-world deployments (Zhang et al., 2006; Dai et al., 2010).

#### 4.1.2. Individual Visual Sensors

Vision-based detection is another prominent method. Extensive effort in this direction has been demonstrated, and some of which (Akagündüz et al., 2017; Ko et al., 2018; Shojaei-Hashemi et al., 2018) show promising performance. Although most cameras are not as portable as wearable devices, they offer other advantages which deem them as decent options depending upon the scenario. Most static RGB cameras are not intrusive and wired hence there is no need to worry about battery limitations. Work on demonstrating viability of vision-based approaches have been demonstrated which makes use of infrared cameras (Mastorakis and Makris, 2014), RGB cameras (Charfi et al., 2012), and RGB-D depth cameras (Cai et al., 2017). One main challenge of vision-based detection is the potential violation of privacy due to the levels of detail that cameras can capture, such as personal information, appearance, and visuals of the living environment.

Further to the information that we report in Table 4, we note that RGB, depth, and infrared cameras are the three main visual sensors used. Moreover, it can be noted that the RGB-D camera (Kinect) is among the most popular vision-based sensor, as 12 out of 22 studies applied it in their work. Nine out of the other 10 studies used RGB cameras including cameras built into smartphones, web cameras, and monocular cameras, while the remaining study used an infrared camera within Kinect, to conduct their experiments.

**Table 4:** Fall detection using individual vision-based devices from 2014 to 2020.

**References**	**Sensor**	**No. subjects (age)**	**Data sets**	**Algorithms**	**Real-time**	**Alarm**
Han et al. (2020)	Web camera	N/A	Simulated	CNN	N/A	N/A
Kong et al. (2019)	Camera (Surveillance)	N/A	Public (Simulated)	CNN	Y	N/A
Ko et al. (2018)	Camera (Smartphone)	N/A	Simulated	Rao-Blackwellized Particle Filtering	N/A	N
Shojaei-Hashemi et al. (2018)	Kinect	40 (10–15)	Public (Simulated)	LSTM	Y	N
Min et al. (2018)	Kinect	4 (N/A), 11 (22–39)	Public (Simulated)	SVM	Y	N
Ozcan et al. (2017)	Web camera	10 (24–31)	Simulated	Relative-entropy-based	N/A	N/A
Akagündüz et al. (2017)	Kinect	10 (N/A)	Public (Simulated) SDU (2011)	Silhouette	N/A	N
Adhikari et al. (2017)	Kinect	5 (19–50)	Simulated	CNN	N/A	N
Ozcan and Velipasalar (2016)	Camera (Smartphone)	10 (24–31)	Simulated	Threshold/ML	N/A	N/A
Senouci et al. (2016)	Web Camera	N/A	Simulated	SVM	Y	Y
Amini et al. (2016)	Kinect v2	11 (24–31)	Simulated	Adaptive Boosting Trigger, Heuristic	Y	N
Kumar et al. (2016)	Kinect	20 (N/A)	Simulated	SVM	N/A	N
Aslan et al. (2015)	Kinect	20 (N/A)	Public (Simulated)	SVM	N/A	N
Yun et al. (2015)	Kinect	12 (N/A)	Simulated	SVM	N/A	N
Stone and Skubic (2015)	Kinect	454 (N/A)	Public (Simulated+Real)	Decision trees	N/A	N
Bian et al. (2015)	Kinect	4 (24–31)	Simulated	SVM	N/A	N
Chua et al. (2015)	RGB camera	N/A	Simulated	Human shape variation	Y	N
Boulard et al. (2014)	Web camera	N/A	Real	Elliptical bounding box	N/A	N
Feng et al. (2014)	Monocular camera	N/A	Simulated	Multi-class SVM	Y	N
Mastorakis and Makris (2014)	Infrared sensor (Kinect)	N/A	Simulated	3D bounding box	Y	N
Gasparrini et al. (2014)	Kinect	N/A	Simulated	Depth frame analysis	Y	N
Yang and Lin (2014)	Kinect	N/A	Simulated	Silhouette	N/A	N

Static RGB cameras are the most widely used sensors within the vision-based fall detection research conducted before 2004, although the accuracies of RGB camera-based detection systems vary drastically due to environmental conditions, such as illumination changes—which often results in limitations during the night. Besides, RGB cameras are inherently likely to have a higher false alarm rate because some deliberate actions like lying on the floor, sleeping or sitting down abruptly are not easily distinguished by frames captured by RGB cameras. With the launch of the Microsoft Kinect, which consists of an RGB camera, a depth sensor, and a multi-array microphone, it stimulated a trend in 3D data collection and analysis, causing a shift from RGB to RGB-D cameras. Kinect depth cameras took the place of the traditional RGB cameras and became the second popular sensors in the field of fall detection after 2014 (Xu et al., 2018).

In the last years, we are seeing an increased interest in the use of wearable cameras for the detection of falls. For instance, Ozcan and Velipasalar (2016) tried to exploit the cameras on smartphones. Smartphones were attached to the waists of subjects and their inbuilt cameras were used to record visual data. Ozcan et al. (2017) investigated how web cameras (e.g., Microsoft LifeCam) attached to the waists of subjects can contribute to fall detection. Although both approaches are not yet practical to be deployed in real applications, they show a new direction, which combines the advantages of wearable and visual sensors.

Table 4 reports the work conducted for individual vision-based sensors. The majority of research still makes use of simulated data. Only two studies use real world data; the one by Boulard et al. (2014) has actual fall data and the other by Stone and Skubic (2015) has mixed data, including 9 genuine falls and 445 simulated falls by trained stunt actors. In contrast to the real data sets from the work of Klenk et al. (2016) collected by wearable devices, there are few purely genuine data sets collected in real life scenarios using individual visual sensors.

#### 4.1.3. Individual Ambient Sensors

The ambient sensor provides another non-intrusive means of fall detection. Sensors like active infrared, RFID, pressure, smart tiles, magnetic switches, Doppler Radar, ultrasonic, and microphone are used to detect the environmental changes due to falling as shown in Table 5. It provides an innovative direction in this field, which is passive and pervasive detection. Ultra-sonic sensor network systems are one of the earliest solutions in fall detection systems. Hori et al. (2004) argues that one can detect falls by putting a series of spatially distributed sensors in the space where elderly persons live. In Wang et al. (2017a,b), a new fall detection approach which uses ambient sensors is proposed. It relies on Wi-Fi which, due to its non-invasive and ubiquitous characteristics, is gaining more and more popularity. However, the studies by Wang et al. (2017a,b) are limited in terms of multi-person detection due to their classifiers not being robust enough to distinguish new subjects and environments. In order to tackle this issue, other studies have developed more sophisticated methods. These include the Aryokee (Tian et al., 2018) and FallDeFi (Palipana et al., 2018) systems. The Aryokee system is ubiquitous, passive and uses RF-sensing methods. Over 140 people were engaged to perform 40 kinds of activities in different environments for the collection of data and a convolutional neural network was utilized to classify falls. Palipana et al. (2018) developed a fall detection technique named FallDeFi, which is based on WiFi signals as the enabling sensing technology. They provided a system applying time-frequency of WiFi Channel State Information (CSI) and achieved above 93% average accuracy.

**Table 5:** Fall detection using individual ambient devices from 2014 to 2020.

**References**	**Sensor**	**No. subjects (age)**	**Data sets**	**Algorithms**	**Real-time**	**Alarm**
Huang et al. (2019)	Vibration	12 (19-29)	Simulated	HMM	Y	N/A
Hao et al. (2019)	WiFi	N/A	Simulated	SVM	Y	N/A
Tian et al. (2018)	FMCW radio	140 (N/A)	Simulated	CNN	Y	N/A
Palipana et al. (2018)	WiFi	3 (27-30)	Simulated	SVM	Y	N/A
Wang et al. (2017a)	WiFi	6 (21-32)	Simulated	SVM	Y	N/A
Wang et al. (2017b)	WiFi	N/A	Simulated	SVM, Random Forests	N/A	N/A

RF-sensing technologies have also been widely applied to other recognition activities beyond fall detection (Zhao et al., 2018; Zhang et al., 2019) and even for subtle movements. Zhao et al. (2018) studied human pose estimation with multiple persons. Their experiment showed that RF-pose has better performance under occlusion. This improvement is attributable to the ability of their method to estimate the pose of the subject through a wall, something that visual sensors fail to do. Further research on RF-sensing was conducted by Niu et al. (2018) with applications to finger gesture recognition, human respiration and chins movement. Their research can be potentially used for applications of autonomous health monitoring and home appliances control. Furthermore, Zhang et al. (2019) used an RF-sensing approach in the proposed system WiDIGR for gait recognition. Guo et al. (2019) claimed that RF-sensing is drawing more attention which can be attributed to being device-free for users, and in contrast to RGB cameras it can work under low light conditions and occlusions.

#### 4.1.4. Subjects

For most research groups there is not enough time and funding to collect data continuously within several years to study fall detection. Due to the rarity of genuine data in fall detection and prediction, Li et al. (2013) have started to hire stunt actors to simulate different kinds of fall. There are also many data sets of falls which are simulated by young healthy students as indicated in the studies by Bourke et al. (2007) and Ma et al. (2014). For obvious reasons elderly subjects cannot be engaged to perform the motion of falls for data collection. For most of the existing data sets, falls are simulated by young volunteers who perform soft falls under the protection of soft mats on the ground. Elderly subjects, however, often have totally different behavior due to less control over the speed of the body. One potential solution could include simulated data sets created using physics engines, such as OpenSim. Previous research (Mastorakis et al., 2007, 2018) have shown that simulated data from OpenSim contributed to an increase in performance to the resulting models. Another solution includes online learning algorithms which adapt to subjects who were not represented in the training data. For instance, Deng et al. (2014) applied the Transfer learning reduced Kernel Extreme Learning Machine (RKELM) approach and showed how they can adapt a trained classifier—based on data sets collected by young volunteers—to the elderly. The algorithm consists of two parts, namely offline classification modeling and online updating modeling, which is used to adapt to new subjects. After the model is trained by labeled training data offline, unlabeled test samples are fed into the pre-trained RKELM classifier and obtain a confidence score. The samples that obtain a confidence score above a certain threshold are used to update the model. In this way, the model is able to adapt to new subjects gradually when new samples are received from new subjects. Namba and Yamada (2018a,b) demonstrated how deep reinforcement learning can be applied to assisting mobile robots, in order to adapt to conditions that were not present in the training set.

### 4.2. Local Communication Layer (LCL) of Individual Sensors

There are two components which are involved with communication within such systems. Firstly, data collected from different smart sensors are sent to local computing facilities or remote cloud computing. Then, after the final decision is made by these computing platforms, instructions and alarms are sent to appointed caregivers for immediate assistance (El-Bendary et al., 2013).

Protocol of data communication is divided into two categories, namely wireless and wired transmission. For the former, transmission protocols include Zigbee, Bluetooth, Wifi, WiMax, and Cellular network.

Most of the studies that used individual wearable sensors deployed commercially available wearable devices. In those cases, data was communicated by transmission modules built in the wearable products, using mediums such as Bluetooth and cellular networks. In contrast to detection systems using wearable devices, most static vision- and ambient-based studies are connected to smart gateways by wired connections. These approaches are usually applied as static detection methods, so a wired connection is a better choice.

### 4.3. Information Processing Layer (IPL) of Individual Sensors

#### 4.3.1. Detection Using Threshold-Based and Data-Driven Algorithms

**Figure 5:**
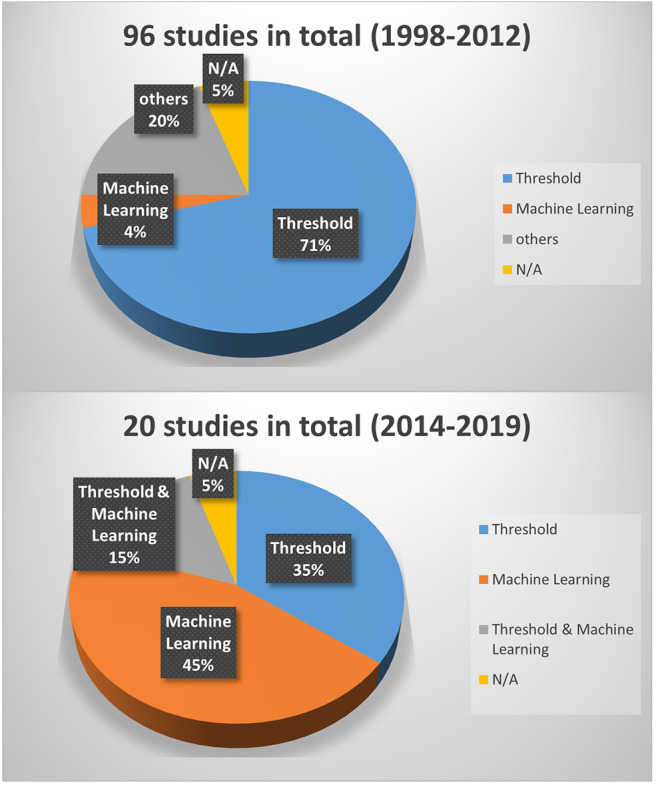
Different types of methods used in fall detection using individual wearable sensors in the period 1998–2012 based on the survey of Schwickert et al. (2013) and in the period 2014–2020 based on our survey. The term “others” refers to traditional methods that are neither based on threshold nor on machine learning, and the term “N/A” stands for not available and refers to studies whose methods are not clearly defined.

Threshold-based and data-driven algorithms (including machine learning and deep learning) are the two main approaches that have been used for fall detection. Threshold-based approaches are usually used for data coming from individual sensors, such as accelerometers, gyroscopes, and electromyography. Their decisions are made by comparing measured values from concerned sensors to empirically established threshold values. Data driven approaches are more applicable for sensor fusion as they can learn non-trivial non-linear relationships from the data of all involved sensors. In terms of the algorithms used to analyze data collected using wearable devices, Figure 5 demonstrates that there is a significant shift to machine learning based approaches, in comparison to the work conducted between 1998 and 2012. From papers presented between 1998 and 2012, threshold-based approaches account for 71%, while only 4% applied machine learning based methods (Schwickert et al., 2013). We believe that this shift is due to two main reasons. First, the rapid development of affordable sensors and the rise of the Internet-of-Things made it possible to more easily deploy multiple sensors in different applications. As mentioned above the non-linear fusion of multiple sensors can be modeled very well by machine learning approaches. Second, with the breakthrough of deep learning, threshold-based approaches have become even less preferable. Moreover, different types of machine learning approaches have been explored, namely, Bayesian networks, rule-based systems, nearest neighbor-based techniques, and neural networks. These data-driven approaches (Gharghan et al., 2018) show better accuracy and they are more robust in comparison to threshold-based methods. Notable is the fact that data-driven approaches are more resource hungry than threshold-based methods. With the ever advancement of technology, however, this is not a major concern and we foresee that more effort will be invested in this direction.

#### 4.3.2. Detection Using Deep Learning

Traditional machine learning approaches determine mapping functions between extracted handcrafted features from raw training data and the respective output labels (e.g., no fall or fall, to keep it simple). The extraction of handcrafted features requires domain expertise and are, therefore, limited to the knowledge of the domain experts. Though such a limitation is imposed, literature shows that traditional machine learning, based on support vector machines, hidden Markov models, and decision trees are still very active in the field of fall detection that uses individual wearable non-visual or ambient sensors (e.g., accelerometer) (Wang et al., 2017a,b; Chen et al., 2018; Saleh and Jeannès, 2019; Wu et al., 2019). For visual sensors the trend has been moving toward deep learning for convolutional neural networks (CNN) (Adhikari et al., 2017; Kong et al., 2019; Han et al., 2020), or LSTM (Shojaei-Hashemi et al., 2018). Deep learning is a sophisticated learning framework that besides the mapping function (as mentioned above and used in traditional machine learning), it also learns the features (in a hierarchy fashion) that characterize the concerned classes (e.g., falls and no falls). This approach has been inspired by the visual system of the mammalian brain (LeCun et al., 2015). In computer vision applications, which take as input images or videos, deep learning has been established as state-of-the-art. In this regard, similar to other computer vision applications, fall detection approaches that rely on vision data have been shifting from traditional machine learning to deep learning in recent years.

#### 4.3.3. Real Time and Alarms

Real-time is a key feature for fall detection systems, especially for commercial products. Considering that certain falls can be fatal or detrimental to the health, it is crucial that the deployed fall detection systems have high computational efficiency, preferably operating in (near) real-time. Below, we comment how the methods proposed in the reviewed literature fit within this aspect.

The percentage of studies applying real-time detection by static visual sensors are lower than that of wearable devices. For the studies using wearable devices, Table 3 illustrates that six out of 20 studies that we reviewed can detect falls and send alarms. There are, however, few studies which demonstrate the ability to process data and send alerts in real-time for work conducted using individual visual sensors. Based on Table 4, one can note that although 40.9% (nine out of 22) of the studies claim that their systems can be used in real-time only one study showed that an alarm can actually be sent in real-time. The following are a couple of reasons why a higher percentage of vision-based systems can not be used in real time. Firstly, visual data is much larger and, therefore, its processing is more time consuming than that of one dimensional signals coming from non-vision-based wearable devices. Secondly, most of the work using vision sensors conducted their experiments with off-line methods, and modules like data transmission were not involved.

##### 4.3.3.1. Summary

- For single-sensor-based fall detection systems most of the studies used data sets that include simulated falls by young and healthy volunteers. Further work is needed to establish whether such simulated falls can be used to detect genuine falls by the elderly.
- The types of sensors utilized in fall detection systems have changed in the past 6 years. For individual wearable sensors, accelerometers are still the most frequently deployed sensors. Static vision-based devices shifted from RGB to RGB-D cameras.
- Data-driven machine learning and deep learning approaches are gaining more popularity especially with vision-based systems. Such techniques may, however, be heavier than threshold-based counterparts in terms of computational resources.
- The majority of proposed approaches, especially those that rely on vision-based sensors, work in offline mode as they cannot operate in real-time. While such methods can be effective in terms of detection, their practical use is debatable as the time to respond is crucial.

## 5. Sensor Fusion by Sensor Network

### 5.1. Physiological Sensing Layer (PSL) Using Sensor Fusion

#### 5.1.1. Sensors Deployed in Sensor Networks

**Figure 6:**
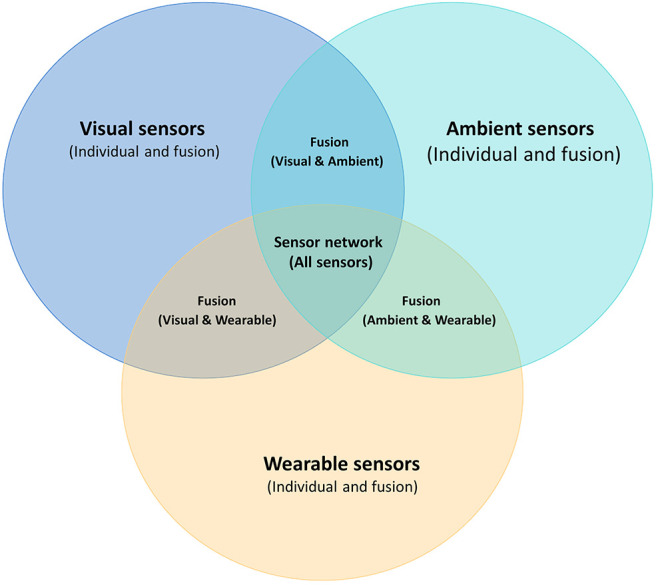
Different kinds of individual sensors and sensor networks, including vision-based, wearable, and ambient sensors, along with sensor fusion.

In terms of sensor fusion, there are two categories, typically referred to as homogeneous and heterogeneous which take input from three types of sensors, namely wearable, visual, ambient sensors, as shown in Figure 6. Sensor fusion involves using multiple and different signals coming from various devices, which may for instance include, accelerometer, gyroscope, magnetometer, and visual sensors, among others. This is all done to complement the strengths of all devices for the design and development of more robust algorithms that can be used to monitor the health of subjects and detect falls (Spasova et al., 2016; Ma et al., 2019).

For the visual detection based approaches, the fusion of signals coming from RGB (Charfi et al., 2012), and RGB-D depth cameras along with camera arrays have been studied (Zhang et al., 2014). They showed that such fusion provides more viewpoints of detected locations, and improves the stability and robustness by decreasing false alarms due to occluded falls (Auvinet et al., 2011).

Li et al. (2018) combined accelerometer data from smartphones and Kinect depth data as well as smartphone camera signals. Liu et al. (2014) and Yazar et al. (2014) fused data from infrared sensors with ambient sensors, and data from doppler and vibration sensors separately. Among them, accelerometers and depth cameras (Kinect) are most frequently studied due to their low costs and effectiveness.

#### 5.1.2. Sensor Networks Platform

Most of the existing IoT platforms, such as Microsoft Azure IoT, IBM Watson IoT Platform, and Google Cloud Platform, have not been used in the deployment of fall detection approaches by sensor fusion. In general, research studies on fall detection using sensor fusion are carried out by offline methods and decision fusion approaches. Therefore, in such studies, there is no need for data transmission and storage modules. From Tables 6, 7, one can also observe that most of the time researchers applied their own workstations or personal computers as their platforms, as there was no need for the integration of sensors and real-time analysis in terms of fall detection in off-line mode.

**Table 6:** Fall detection by fusion of wearable sensors from 2014 to 2020.

**Fusion within wearable sensors**							
**References**	**Sensor**	**No. subjects (age)**	**Data sets**	**Algorithms**	**Real-time (Alarm)**	**Fusion method**	**Platforms**
Kerdjidj et al. (2020)	Accelerometer, Gyroscope	17 (N/A)	Simulated	Compressive sensing	Y (N/A)	Feature fusion	N/A
Xi et al. (2020)	Electromyography, Plantar Pressure	12 (23–27)	Simulated	FMMNN, DPK-OMELM	Y (Y)	Feature fusion	N/A
Chelli and Pätzold (2019)	Accelerometer, Gyroscope	30 (N/A)	Public (Simulated)	KNN, ANN, QSVM, EBT	Y (N/A)	Feature fusion	N/A
Queralta et al. (2019)	Accelerometer, Gyroscope, Magnetometer	57 (20-47)	Public (Simulated)	LTSM	Y(Y)	Feature fusion	N/A
Gia et al. (2018)	Accelerometer, Gyroscope, Magnetometer	2 (N/A)	N/A	Threshold	Y (Y)	Feature fusion	N/A
de Quadros et al. (2018)	Accelerometer, Gyroscope, Magnetometer	22 (mean = 26.09)	Simulated	Threshold/ML	N/A	Feature fusion	N/A
Yang et al. (2016)	Accelerometer, Gyroscope, Magnetometer	5 (N/A)	Simulated	SVM	Y (Y)	Feature fusion	PC
Pierleoni et al. (2015)	Accelerometer, Gyroscope, Magnetometer	10 (22–29)	Simulated	Threshold	Y (Y)	Feature fusion	ATmega328p (ATMEL)
Nukala et al. (2014)	Accelerometer, Gyroscopes	2 (N/A)	Simulated	ANN	Y (N/A)	Feature fusion	PC
Kumar et al. (2014)	Accelerometer, Pressure sensors, Heart rate monitor	N/A	Simulated	Threshold	Y (Y)	Partial fusion	PC
Hsieh et al. (2014)	Accelerometer, Gyroscope	3 (N/A)	Simulated	Threshold	N/A	Partial fusion	N/A

**Table 7:** Fall detection using fusion of sensor networks from 2014 to 2020.

**References**	**Sensor**	**No. subjects (age)**	**Data sets**	**Algorithms**	**Real-time (Alarm)**	**Fusion method**	**Platforms**
**Fusion within visual sensors and ambient sensors**							
Espinosa et al. (2019)	Two cameras	17 (18-24)	Simulated	CNN	N/A (N)	Feature fusion	N/A
Ma et al. (2019)	RGB camera, Thermal camera	14 (N/A)	Simulated	CNN	N/A (N)	Partial fusion	N/A
Spasova et al. (2016)	Web Camera, Infrared sensor	5 (27-81)	Simulated	SVM	Y (Y)	Partial fusion	A13-OlinuXino
**Fusion within different kinds of individual sensors**							
Mart́ınez-Villaseñor et al. (2019)	Accelerometer, Gyroscope, Ambient light, Electroencephalograph, Infrared sensors, Web cameras	17 (18–24)	Simulated	Random Forest, SVM, ANN, kNN, CNN	Feature fusion	N/A	N/A
Li et al. (2018)	Accelerometer (smartphone), Kinect	N/A	Simulated	SVM, Threshold	Y (N/A)	Decision fusion	N/A
Daher et al. (2017)	Force sensors, Accelerometers	6 (N/A)	Simulated	Threshold	N (N/A)	Decision fusion	N/A
Ozcan and Velipasalar (2016)	Camera (smartphone), Accelerometer	10 (24 -30)	Simulated	Histogram of oriented gradients	Y (Y)	Decision fusion	N/A
Kwolek and Kepski (2016)	Accelerometer, Kinect	5 (N/A)	Simulated	Fuzzy logic	Y (Y)	Feature fusion, Partial fusion	PandaBoard ES
Sabatini et al. (2016)	Barometric altimeters, Accelerometer, Gyroscope	25 (mean = 28.3)	Simulated	Threshold	N/A (N)	Feature fusion	N/A
Chen et al. (2015)	Kinect, Inertial sensor	12 (23–30)	Public Simulated Ofli et al. (2013)	Collaborative representation,	N/A (N)	Feature fusion	N/A
Gasparrini et al. (2015)	Kinect v2, Accelerometer	11 (22-39)	Simulated	Threshold	N (N/A)	Data fusion	N/A
Kwolek and Kepski (2014)	Accelerometer, Kinect	5 (N/A)	Public (Simulated) URF (2014)	SVM, k-NN	Y (Y)	Partial fusion	PandaBoard ES
Kepski and Kwolek (2014)	Accelerometer, Kinect	30 (under 28)	Simulated	Alogorithms	Y (N)	Partial fusion	PandaBoard
Liu et al. (2014)	Passive infrared sensor, Doppler radar sensor	454 (N/A)	Simulated + Real life	SVM	N/A (N)	Decision fusion	N/A
Yazar et al. (2014)	Passive infrared sensors, Vibration sensor	N/A	Simulated	Threshold, SVM	N/A (N)	Decision fusion	N/A

Some works, such as those in Kwolek and Kepski (2014), Kepski and Kwolek (2014), and Kwolek and Kepski (2016), applied low-power single-board computer development platforms running in Linux, namely PandaBoard, PandaBoard ES, and A13-OlinuXino. A13-OlinuXino is an ARM-based single-board computer development platform, which runs Debian Linux distribution. PandaBoard ES, which is the updated version of PandaBoard, is a single-board computer development platform running at Linux. The PandaBoard ES can run different kinds of Linux-based operating systems, including Android and Ubuntu. It consists of 1 GB of DDR2 SDRAM, dual USB 2.0 ports as well as wired 10/100 Ethernet along with wireless Ethernet and Bluetooth connectivity. Linux is well-known for real-time embedded platforms since it provides various flexible inter-process communication methods, which is quite suitable for fall detection using sensor fusion.

In the research by Kwolek and Kepski (2014, 2016), wearable devices and Kinect were connected to the Pandaboard through Bluetooth and cable, separately. Firstly, data was collected by accelerometers and Kinect sensors, individually, which was then transmitted and stored in a memory card. The procedure of data transmission is asynchronous since there are different sampling rates for accelerometers and Kinect. Finally, all data was grouped together and processed by classification models that detected falls. The authors reported high accuracy rates but could not compare with other approaches since there is no benchmark data set.

Spasova et al. (2016) applied the A13-OlinuXino board as their platform. A standard web camera was connected to it via USB and an infrared camera was connected to the development board via I2C (Inter-Integrated Circuit). Their experiment achieved excellent performance with over 97% sensitivity and specificity. They claim that their system can be applied in real-time with hardware of low-cost and open source software platform.

Despite the available platforms mentioned above, the majority of fall detection studies trained their models in an offline mode with a single sensor on personal computers. The studies in Kwolek and Kepski (2014), Kepski and Kwolek (2014), Kwolek and Kepski (2016), and Spasova et al. (2016) utilized single-board computer platforms in their experiments to demonstrate the efficacy of their approaches. The crucial aspects of scalability and efficiency were not addressed and hence it is difficult to speculate the appropriateness of their methods in real-world applications. We believe that the future trend is to apply an interdisciplinary approach that deploys the data analysis modules on mature cloud platforms, which can provide a stable and robust environment while meeting the exploding demands of commercial applications.

#### 5.1.3. Subjects and Data Sets

Although some groups devoted their efforts to acquire data of genuine falls, most researchers used data that contained simulated falls. We know that monitoring the lives of elderly people and waiting to capture real falls is very sensitive and time consuming. Having said that though, with regards to sensor fusion by wearable devices, there have been some attempts which have tried to build data sets of genuine data in real life. FARSEEING (Fall Repository for the design of Smart and self-adaptive Environments prolonging Independent living) is one such data set (Klenk et al., 2016). It is actually the largest data set of genuine falls in real life, and is open to public research upon request on their website. From 2012 to 2015, more than 2,000 volunteers have been involved, and more than 300 real falls have been collected under the collaboration of six institutions^3^.

As for the fusion by visual sensors and the combination of other non-wearable sensors, it becomes quite hard to acquire genuine data in real life. There was one group which tried to collect real data by visual sensors, but only nine real falls by elderly (Demiris et al., 2008) were captured during several years. The availability of only nine falls is too limited to train a meaningful model. As an alternative, Stone and Skubic (2015) hired trained stunt actors to simulate different kinds of falls and made a benchmark data set with 454 falls including 9 real falls by elderly.

### 5.2. Local Communication Layer (LCL) Using Sensor Fusion

Data transmission for fall detection using sensor networks can be done in different ways. In particular, Bluetooth (Pierleoni et al., 2015; Yang et al., 2016), Wi-Fi, ZigBee (Hsieh et al., 2014), cellular network using smart phones (Chen et al., 2018) and smart watches (Kao et al., 2017), as well as wired connection have all been explored. In studies that used wearable devices, most of them applied wireless methods, such as Bluetooth, which allowed the subject to move unrestricted.

Currently, when it comes to wireless sensors, Bluetooth has become probably the most popular communication protocol and it is widely used in existing commercial wearable products such as Shimmer. In the work by Yang et al. (2016), data is transmitted to a laptop in real-time by a Bluetooth module that is built in a commercial wearable device named Shimmer 2R. The sampling frame rate can be customized, and they chose to work with the 32-Hz sampling rate instead of the default sampling rate of 51.2-Hz. At high sampling frequencies, packet loss can occur and higher sampling rate also means higher energy consumption. Bluetooth is also applied to transmit data in non-commercial wearable devices. For example, Pierleoni et al. (2015) customized a wireless sensor node, where sensor module, micro-controller, Bluetooth module, battery, mass-storage unit, and wireless receiver were integrated within a prototype device of size 70–45–30 mm. Zigbee was used to transmit data in the work by Hsieh et al. (2014). In Table 8, we compare different kinds of wireless communication protocols.

**Table 8:** Comparison of different kinds of communication protocol.

**Protocol**	**Zigbee**	**Bluetooth**	**WiFi**	**WiMax**	**Cellular network**
Range	100 m	10 m	5 km	15 km	10–50 km
Data rate	250–500 kbps	1–3 Mbps	1–450 Mbps	75 Mbps	240 kbps
Band-width	2.4 GHz	2.4 GHz	2.4, 3.7, and 5 GHz	2.3, 2.5, and 3.5 GHz	824–894 MHz/1,900 MHz
Energy consumption	Low	Medium	High	N/A	N/A

As for the data transmission using vision-based and ambient-based approaches, wired options are usually preferred. In the work by Spasova et al. (2016), a standard web camera was connected to an A13-OlinuXino board via USB and an infrared camera was connected to the development board via I2C (Inter-Integrated Circuit). Data and other messages were exchanged within the smart gateways through the internet.

For sensor fusion using different types of sensors, both wireless and cabled methods were utilized because of data variety. In the work by Kwolek and Kepski (2014, 2016), wearable devices and Kinect were connected to the Pandaboard through Bluetooth and cable, separately. Kinect was connected to a PC using USB interface and smart phones were connected by wireless methods (Li et al., 2018). These two types of sensor, smartphone and Kinect, were first used separately to monitor the same events and the underlying methods that processed their signals sent their output to a Netty server through the Internet where another method was used to fuse the outcomes of both methods to come to a final decision of whether the involved individual has fallen or not.

In the studies by Kwolek and Kepski (2014, 2016), accelerometers and Kinect cameras were connected to a pandaboard through Bluetooth and USB connections. Then, the final decision was made based on the data collected from the two sensors.

### 5.3. Information Processing Layer (IPL) Using Sensor Fusion

#### 5.3.1. Methods of Sensor Fusion

**Figure 7:**
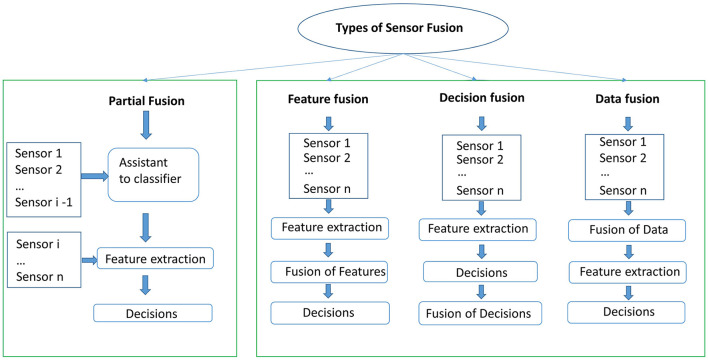
Four kinds of sensor fusion methods including partial fusion, feature fusion, decision fusion, and data fusion. Partial fusion means that a subset of sensors are deployed to make decisions, while the other types of fusion techniques use all sensors as input.

Speaking of the fusion of different sensors, there are several criteria to group them. Yang and Yang (2006) and Tsinganos and Skodras (2018) grouped them into three categories, namely direct data fusion, feature fusion, and decision fusion. We divide sensor fusion techniques into four groups as shown in Figure 7, which we refer to as fusion with partial sensors, direct data fusion, feature fusion, and decision fusion.

For the partial fusion, although multiple sensors are deployed, only one sensor is used to take the final decision, such as the work by Ma et al. (2019). They used an RGB and a thermal camera to conduct their experiments, with the thermal camera being used only for the localization of faces. Falls were eventually detected only based on the data collected from the regular RGB cameras. A similar approach was applied by Spasova et al. (2016), where an infrared camera was deployed to confirm the presence of the subject and the data produced by the RGB camera was used to detect falls. There are also other works that used wearable devices that deployed the sensors at different stages. For instance, in (Kepski and Kwolek, 2014; Kwolek and Kepski, 2014) a fall detection system was built by utilizing a tri-axial accelerometer and an RGB-D camera. The accelerometer was deployed to detect the motion of the subject. If the measured signal exceeded a given threshold then the Kinect was activated to capture the ongoing event.

The second approach of sensor fusion is known as feature fusion. In such an approach, feature extraction takes places on signals that come from different sensors. Then all features are merged into long feature vectors and used to train classification models. Most of the studies that we reviewed applied feature fusion for wearable-based fall detection systems. Many commercial products of wearable devices, sensors like accelerometers, gyroscope, magnetometer are built in one device. Data from these sensors is homogeneous synchronous with the same frequency and transmitted with built-in wireless modules. Having signals producing data with the synchronized frequency simplifies the fusion of data. Statistical features, such as mean, maximum, standard deviation, correlation, spectral entropy, spectral, sum vector magnitude, the angle between y-axis and vertical direction, and differential sum vector magnitude centroid can be determined from the signals of accelerometers, magnetometers, and gyroscopes, and used as features to train a classification model that can detect different types of falls (Yang et al., 2016; de Quadros et al., 2018; Gia et al., 2018).

Decision fusion is the third approach, where a chain of classifiers is used to come to a decision. A typical arrangement is to have a classification model that takes input from one type of sensor, another model that takes input from another sensor, and in turn the outputs of these two models are used as input to a third classification model that takes the final decision. Li et al. (2018) explored this approach with accelerometers embedded in smart phones and Kinect sensors. Ozcan and Velipasalar (2016) deployed an accelerometer and an RGB camera for the detection of falls. Different sensors, such as accelerometer, RGB and RGB-D cameras were deployed in these studies. Decisions are made separately based on the individual sensors, and then the final decision is achieved by combining the individual sensors.

The final approach is data fusion. This is achieved by first fusing the data from different sensors and perform feature extraction from the fused data. This is in contrast to feature fusion where data from these sensors is homogeneous synchronous with the same frequency. Data fusion can be applied to different sensors with different sampling frequency and data characteristics. Data from various sensors can be synchronized and combined directly for some sensors of different types. Because of the difference in sampling rate between the Kinect camera and wearable sensors, it is challenging to conduct feature fusion directly. In order to mitigate this difficulty, the transmission and exposure times of the Kinect camera are adapted to synchronize the RGB-D data with that of wearable sensors by an *ad-hoc* acquisition software, as was done by Gasparrini et al. (2015).

Ozcan and Velipasalar (2016) used both partial and feature fusion. They divided the procedure in two stages. In the first stage, only the accelerometer was utilized to indicate a potential fall, then the Kinect camera activates after the accelerometer flagged a potential fall. Features from both the Kinect camera and accelerometer were then extracted to classify activities of fall or non-fall in the second stage.

#### 5.3.2. Machine Learning, Deep Learning, and Deep Reinforcement Learning

In terms of fall detection techniques based on wearable sensor fusion, the explored methods include threshold-based, traditional machine learning, and deep learning. The latter two are the most popular due to their robustness. The research by Chelli and Pätzold (2019) applied both traditional machine learning [kNN, QSVM, Ensemble Bagged Tree (EBT)] and deep learning. Their experiments were divided into two parts, namely activity recognition and fall detection. For the former, their experiments showed that traditional machine learning and deep learning outperformed other approaches, which showed 94.1 and 93.2% accuracy, respectively. Queralta et al. (2019) applied a long short-term memory (LSTM) approach, where wearable nodes including accelerometer, gyroscope, and magnetometer were embedded in a low power wide area network, with combined edge and fog computing. The LSTM algorithm is a type of recurrent neural network aimed at solving long sequence learning tasks. Their system achieved an average recall of 95% while providing a real-time solution of fall detection running on cloud platforms. Another example is the work by Nukala et al. (2014) who fused the measurements of accelerometers and gyroscopes and applied an Artificial Neural Network (ANN) for the modeling of fall detection.

As for visual sensor based fusion techniques, the limited studies that were included in our survey applied either traditional machine learning or deep learning (Espinosa et al., 2019; Ma et al., 2019) approaches. Fusion of multiple visual sensors from a public data set was presented by Espinosa et al. (2019), where a 2D CNN was trained to classify falls during daily life activities.

Another approach is reinforcement learning (RL), which is a growing branch in machine learning, and is gaining popularity in the fall detection field as well. Deep reinforcement learning (DRL) combines the advantages of deep learning and reinforcement learning, and has already shown its benefits in fall prevention (Namba and Yamada, 2018a,b; Yang, 2018) and fall detection (Yang, 2018). Namba and Yamada (2018a) proposed a fall risk prevention approach by assisting robots for the elderly living independently. Images and movies with the location information of accidents were collected. Most conventional machine learning and deep learning methods are, however, challenged when the operational environment changes. This is due to their data-driven nature that allows them to learn how to become robust mostly in the same environments where they were trained.

#### 5.3.3. Data Storage and Analysis

Typical data storage devices include SD cards, local storage on the integration device, or remote storage on the cloud. For example, some studies used the camera and accelerometer in smartphones, and stored the data on the local storage of the smarphones (Ozcan and Velipasalar, 2016; Shi et al., 2016; Medrano et al., 2017). Other studies applied off-line methods and stored data in their own computer, and could be processed at a later stage. Alamri et al. (2013) argue that sensor-cloud will become the future trend because cloud platforms can be more open and more flexible than local platforms, which have limited local storage and processing power.

### 5.4. User Application Layer (UAL) of Sensor Fusion

Due to the rapid development of miniature bio-sensing devices, there has been a booming development of wearable sensors and other fall detection modules. Wearable modules, such as Shimmer, embedded with sensing sensors, communication protocols, and sufficient computational ability are available as affordable commercial products. For example, some wearable-based applications have been applied to the detection of falls and for monitoring health, in general. The target of the wearable devices is to wear and forget. Taking as an example the electronic skins (e-skins) that adhere to the body surface, clothing-based or accessory-based devices where proximity is sufficient. To fulfill the target of wearing and forgetting, many efforts have been put into the study of wearable systems, such as the My Heart project (Habetha, 2006), the Wearable Health Care System (WEALTHY) project (Paradiso et al., 2005), the Medical Remote Monitoring of clothes (MERMOTH) project (Luprano, 2006), and the project by Pandian et al. (2008). Some wearable sensors are also developed specifically to address fall detection. Shibuya et al. (2015) used a wearable wireless gait sensor for the detection of falls. More and more research work use existing commercial wearable products, which includes function of data transmission and sending alarms when falls are detected.

#### 5.4.1. Summary

- Due to the sampling frequency and data characteristic, there are two main categories for sensor fusion. As shown in Tables 6, 7, studies by sensor fusion are divided into fusion by sensor from the same category (e.g., fusion of wearable sensors, fusion of visual sensors, and fusion of ambient sensors) and fusion of sensors from different categories.
- Subjects in fall detection studies using sensor networks are still young and healthy volunteers, which is similar to that of individual sensors. Only one research adopted mixed data with simulated and genuine data.
- More wearable-based approaches are embedded with IoT platforms than that of vision-based approaches because data transmission and storage modules are built in existing commercial products.
- For the research combining sensors from different categories, the combination of accelerometer and Kinect camera is the most popular method.
- Partial fusion, data fusion, feature fusion, and decision fusion are four main methods of sensor fusion. Among them, feature fusion is the most popular approach, followed by decision fusion. For fusion using non-vision wearable sensors, most of the studies that we reviewed applied feature fusion, while decision fusion is the most appealing one for fusing sensors from different categories.

## 6. Security and Privacy

Because data generated by autonomous monitoring systems are security-critical and privacy-sensitive, there is an urgent demand to protect user's privacy and prevent these systems from being attacked. Cyberattacks on the autonomous monitoring systems may cause physical or mental damages and even threaten the lives of subjects under monitoring.

### 6.1. Security

In this survey we approached the systems of fall detection from different layers, including Physiological Sensing Layer (PSL), Local Communication Layer (LCL), Information Processing Layer (IPL), Internet Application Layer (IAL), and User Application Layer (UAL). Every layer faces security issues. For instance, information may leak in the LCL during data transmission, along with potential vulnerabilities with cloud storage and processing facility. Based on the literature that we report in Tables 3–7, most of the studies in the field of fall detection do not address security matters. Only few studies (Edgcomb and Vahid, 2012; Mastorakis and Makris, 2014; Ma et al., 2019) take privacy into consideration. Because of the distinct characteristics of wired and wireless transmission, it is still an open problem to find a comprehensive security protocol which can cover the security issues in both wired and wireless data transmission and storage (Islam et al., 2015).

### 6.2. Privacy

As mentioned above, privacy is one of the most important issue for users of autonomous health monitoring systems. Methods to protect privacy are dependent on the type of sensor used. Not all sensors tend to suffer from the issues of privacy equally. For example, vision-based sensors, like RGB cameras, are more vulnerable than wearable sensors, such as accelerometers, in terms of privacy. In the case of a detection system that uses only wearable sensors, problems of privacy are not as critical as systems involved with visual sensors.

In order to address the privacy concerns associated with RGB cameras some researchers proposed to mitigate them by blurring and distorting the appearances as post-processing steps in the application layer (Edgcomb and Vahid, 2012). An alternative way is to address the privacy issue in the design stage, as suggested by Ma et al. (2019). They investigated an optical level anonymous image sensing system. A thermal camera was deployed to locate faces and an RGB camera was used to detect falls. The location of the subject's face was used to generate a mask pattern on a spatial light modulator to control the light entering the RGB camera. Faces of subjects were blurred by blocking the visible light rays using the mask pattern on the spatial light modulator.

The infrared camera is another sensor which could protect the privacy of subjects. Mastorakis and Makris (2014) investigated an infrared camera built in a Kinect sensor. It only captures the thermal distribution of subjects and there is no information on the subject's appearance and living environment involved. Other vision-based sensors which could protect privacy are depth cameras. The fact they only capture depth information has made them more popular than RGB cameras.

As for the research of fall detection using sensor networks, different kinds of data are collected when more sensors are involved. Because of more data collection and transfer involved, the whole fall detection system by sensor fusion becomes more complicated and it makes the protection of privacy and security even harder. There is a trade-off between privacy and benefits of autonomous monitoring systems. The aim is to keep improving the algorithms while keeping the privacy and security issues to a minimum. This is the only way to make such systems socially acceptable.

## 7. Projects and Applications Around Fall Detection

Approaches of fall detection evolve from personal emergency response systems (PERS) to intelligent automatic ones. One of the early fall detection systems sends an alarm by the PERS push-button, but it may fail when the concerned person loses consciousness or is too weak to move (Leff, 1997). Numerous attempts have been made to monitor not only falls but also other specific activities in autonomous health monitoring. Many projects have been conducted to develop applications of autonomous health monitoring, including fall detection, prediction, and prevention. Some of the aforementioned studies were promoted as commercial products. Different sensors from wearable sensors, visual sensors, and ambient sensors are deployed as commercial applications for fall detection. Among them, more wearable sensors have been developed as useful applications. For example, a company named Shimmer has developed 7 kinds of wearable sensing products aiming at autonomous health monitoring. One of the products is the Shimmer3 IMU Development Kit. It is a wearable sensor node including a sensing module, data transmission module, receiver, and it has been used by Mahmud and Sirat (2015) and Djelouat et al. (2017). The iLife fall detection sensor is developed by AlertOne^4^, which provides the service of fall detection and one-button alert system. Smartwatch is another commercial solution for fall detection. Accelerometers embedded in smartwatches have been studied to detect falls (Kao et al., 2017; Wu et al., 2019). Moreover, Apple Watch Series 4 and later versions are equipped with the fall detection function, and it can help the consumer to connect to the emergency service. Although there are few specific commercial fall detection products based on RGB cameras, the relevant studies also show a promising future in the field. There are open source solutions provided by Microsoft using Kinect which could detect falls in real time and have the potential to be deployed as commercial products. As for ambient sensors, Linksys Aware apply tri-band mesh WiFi systems to fall detection, and they provide a premium subscription service as a commercial motion detection product. CodeBlue, a Harvard University research project, also focused on developing wireless sensor networks for medical applications (Lorincz et al., 2004). The MIThril project (DeVaul et al., 2003) is a next-generation wearable research platform developed by researchers at the MIT Media Lab. They made their software open source and hardware specifications available to the public.

The Ivy project (Pister et al., 2003) is a sensor network infrastructure from the Berkeley College of Engineering, University of California. The project aims to develop a sensor network system to provide assistance for the elderly living independently. Using a sensor network with fixed sensors and mobile sensors worn on the body, anomalies by the concerned elderly can be detected. Once falls are detected, the system sends alarms to caregivers to respond urgently.

A sensor network was built in 13 apartments in TigerPlace, which is an aging in place for people of retirement in Columbia, Missouri, and continuous data was collected for 3,339 days (Demiris et al., 2008). The sensor network with simple motion sensors, video sensors, and bed sensors that capture sleep restlessness and pulse and respiration levels, were installed in some apartments of 14 volunteers. Activities of 16 elderly people in TigerPlace, whose age range from 67 to 97, were recorded continuously and 9 genuine falls were captured. Based on the data set, Li et al. (2013) developed a sensor fusion algorithm. which achieved low rate of false alarms and a high detection rate.

## 8. Trends and Open Challenges

### 8.1. Trends

#### 8.1.1. Sensor Fusion

There seems to be a general consensus that sensor fusion provides a more robust approach for the detection of elderly falls. The use of various sensors may complement each other in different situations. Thus, instead of relying on only one sensor, which may be unreliable if the conditions are not suitable for that sensor, the idea is to rely on different types of sensor that together can capture reliable data in various conditions. This results in a more robust system that can keep false alarms to a minimum while achieving high precision.

#### 8.1.2. Machine Learning, Deep Learning and Deep Reinforcement Learning

Conventional machine learning approaches have been widely applied in fall detection and activity recognition, and results outperform those of threshold-based methods in studies that use wearable sensors. Deep learning is a subset of machine learning, which is concerned with artificial neural networks inspired by the mammalian brain. Approaches of deep learning are gaining popularity especially for visual sensors and sensor fusion and are becoming the state-of-the-art for fall detection and other activity recognition. Deep reinforcement learning is another promising research direction for fall detection. Reinforcement learning is inspired by the psychological neuro-scientific understandings of humans which can adapt and optimize decisions in a changing environment. Deep reinforcement learning combines advantages of deep learning, and reinforcement learning which can provide alternatives for detection that can adapt to the changing condition without sacrificing accuracy and robustness.

#### 8.1.3. Fall Detection Systems on 5G Wireless Networks

5G is a softwarized and virtualized wireless network, which includes both a physical network and software virtual network functions. In comparison to 4G networks, 5th generation mobile introduces the ability of data transmission with high speed and low latency, which could contribute to the development of fall detection by IoT systems. Firstly, 5G is envisioned to become an important and universal communication protocol for IoT. Secondly, 5G cellular can be used for passive sensing approaches. Different from other kinds of RF-sensing approaches (e.g., WiFi or radar) which are aimed for short-distance indoor fall detection, the 5G wireless network can be applied to both indoor and outdoor scenarios as a pervasive sensing method. This type of network has already been successfully investigated by Gholampooryazdi et al. (2017) for the detection of crowd-size, presence detection, and walking speed, and their experiments showed accuracy of 80.9, 92.8, and 95%, respectively. Thirdly, we expect that 5G as a network is going to become a highly efficient and accurate platform to achieve better performance of anomaly detection. Smart networks or systems powered by 5G IoT and deep learning can be applied not only in fall detection systems, but also in other pervasive sensing and smart monitoring systems which assist elderly groups to live independently with high-quality life.

#### 8.1.4. Personalized or Simulated Data

El-Bendary et al. (2013) and Namba and Yamada (2018b) have proposed to include historical medical and behavioral data of individuals along with sensor data. This allowed the enrichment of the data and consequently to make better informed decisions. This innovative perspective allows a more personalized approach as it uses the health profile of the concerned individual and it has the potential to become a trend also in this field. Another trend could be the way data sets are created to evaluate systems for fall detection. Mastorakis et al. (2007, 2018) applied the skeletal model simulated in Opensim, which is an open-source software developed by Stanford University. It can simulate different kinds of pre-defined skeletal models. They acquired 132 videos of different types of falls, and trained their own algorithms based on those models. The high results that they report indicate that the simulated falls by OpenSim are very realistic and, therefore, effective for training a fall detection model. Physics engines, like Opensim, can simulate customized data based on the height and age of different subjects and it offers the possibility of new directions to detect falls. Another solution, which can potentially address the scarcity of data, is to develop algorithms that can be adapted to subjects that were not part of the original training set (Deng et al., 2014; Namba and Yamada, 2018a,b) as we described in section 4.1.4.

#### 8.1.5. Fog Computing

As to architecture is concerned, Fog computing offers the possibility to distribute different levels of processing across the involved edge devices in a decentralized way. Smart devices that can carry out some processing and that can communicate directly with each other are more attractive for (near) real-time processing as opposed to systems based on cloud computing (Queralta et al., 2019). An example of such smart devices include the Intel® RealSense™ depth camera, which includes a 28 nanometer (nm) processor to compute real-time depth images.

### 8.2. Open Challenges

The topic of fall detection has been studied extensively during the past two decades and many attempts have been proposed. The rapid development of new technologies keeps this topic very active in the research community. Although much progress has been made, there are still various open challenges, which we discuss below.

1. **The rarity of data of real falls**: There is no convincing public data set which could provide a gold standard. Many simulated data sets by individual sensors are available, but it is debatable whether models trained on data collected by young and healthy subjects can be applied to elderly people in real-life scenarios. To the best of our knowledge, only Liu et al. (2014) used a data set with nine real falls along with 445 simulated ones. As for data sets with multiple sensors, the data sets are even scarcer. There is, therefore, an urgent need to create a benchmark data set of data coming from multiple sensors.
2. **Detection in real-time:** The attempts that we have seen in the literature are all based on offline methods that detect falls. While this is an important step, it is time that research starts focusing more on real-time systems that can be applied in the real-world.
3. **Security and privacy**: We have seen little attention to the security and privacy concerned with fall detection approaches. Security and privacy is therefore another topic which to our opinion must be addressed in cohesion with fall detection methods.
4. **Platform of sensor fusion:** It is still a novice topic with a lot of potential. Studies so far have treated this topic to a minimum as they mostly focused on the analytics aspect of the problem. In order to bring solutions closer to the market more holistic studies are needed to develop full information systems that can deal with the management and transmission of data in an efficient, effective and secure way.
5. **Limitation of location:** Some sensors, such as visual ones, have limited capability because they are fixed and static. It is necessary to develop fall detection systems which can be applied to controlled (indoor) and uncontrolled (outdoor) environments.
6. **Scalability and flexibility:** With the increasing number of affordable sensors there is a crucial necessity to study the scalability of fall detection systems especially when inhomogeneous sensors are considered (Islam et al., 2015). There is an increasing demand for scalable fall detection approaches that do not sacrifice robustness or security. When considering cloud-based trends, fall detection modules, such as data transmission, processing, applications, and services, should be configurable and scalable in order to adapt to the growth of commercial demands. Cloud-based systems enable more scalability of health monitoring systems at different levels as the need for resources of both hardware and software level changes with time. Cloud-based systems can add or remove sensors and services with little effort on the architecture (Alamri et al., 2013).

## 9. Summary and Conclusions

In this review we give an account on fall detection systems from a holistic point of view that includes data collection, data management, data transmission, security and privacy as well as applications.

In particular we compare approaches that rely on individual sensors with those that are based on sensor networks with various fusion techniques. The survey provides a description of the components of fall detection and it is aimed to give a comprehensive understanding of physical elements, software organization, working principles, techniques, and arrangement of different components that concern fall detection systems.

We draw the following conclusions.

1. The sensors and algorithms proposed during the past 6 years are very different in comparison to the research before 2014. Accelerometers are still the most popular sensors in wearable devices, while Kinect took the place of the RGB camera and became the most popular visual sensor. The combination of Kinect and accelerometer is turning out to be the most sought after.
2. There is not yet a benchmark data set on which fall detection systems can be evaluated and compared. This creates a hurdle in advancing the field. Although there has been an attempt to use middle-age subjects to simulate falls (Kangas et al., 2008), there are still differences in behavior between the elderly and middle-aged subjects.
3. Sensor fusion seems to be the way forward. It provides more robust solutions in fall detection systems but come with higher computational costs when compared to those that rely on individual sensors. The challenge is therefore to mitigate the computational costs.
4. Existing studies focus mainly on the data analytics aspect and do not give too much attention to IoT platforms in order to build full and stable systems. Moreover, the effort is put on analyzing data in offline mode. In order to bring such systems to the market, more effort needs to be invested in building all the components that make a robust, stable, and secure system that allows (near) real-time processing and that gains the trust of the elderly people.

The detection of elderly falls is an example of the potential of autonomous health monitoring systems. While the focus here was on elderly people, the same or similar systems can be applicable to people with mobility problems. With the ongoing development of IoT devices, autonomous health monitoring and assistance systems that rely on such devices seems to be the key for the detection of early signs of physical and cognitive problems that can range from cardiovascular issues to mental disorders, such as Alzheimer's and dementia.

## Author Contributions

GA and XW conceived and planned the paper. XW wrote the manuscript in consultation with GA and JE. All authors listed in this paper have made a substantial, direct and intellectual contribution to the work, and approved it for publication.

## Conflict of Interest

The authors declare that the research was conducted in the absence of any commercial or financial relationships that could be construed as a potential conflict of interest.

## Footnotes

^1^https://chinapower.csis.org/aging-problem/

^2^https://www.google.com/trends

^3^1. Robert-Bosch Hospital (RBMF), Germany; 2. University of Tübingen, Germany; 3. University of Nürnberg/Erlangen, Germany; 4. German Sport University Cologne, Germany; 5. Bethanien-Hospital/Geriatric Center at the University of Heidelberg, Germany; 6. University of Auckland, New Zealand.

^4^https://www.alert-1.com/
